# Editorial: Discussing structural, systemic and enabling approaches to socio-environmental transformations: Stimulating an interdisciplinary and plural debate within the social sciences

**DOI:** 10.3389/fsoc.2022.968018

**Published:** 2022-08-16

**Authors:** Marco Billi, Cristina Zurbriggen, Daniel Morchain

**Affiliations:** ^1^Department of Rural Management and Innovation, University of Chile, Santiago, Chile; ^2^Center for Climate and Resilience Research, University of Chile, Santiago, Chile; ^3^Transdisciplinary Systemic Research Hub (NEST.R3), Santiago, Chile; ^4^Instituto de Ciencia Política, Facultad de Ciencias Sociales, Universidad de la República, Montevideo, Uruguay; ^5^South American Institute for Resilience and Sustainability Studies (SARAS), Maldonado, Uruguay; ^6^The Nature Conservancy, Arlington, VA, United States; ^7^International Institute for Sustainable Development, Winnipeg, MB, Canada

**Keywords:** transformation, systems theory, socio-ecological resilience, socio-technical transitions, social framing, social learning

In recent decades, increasing calls have been raised at engaging more deeply with the “social” aspects of the evolving interconnection and interdependence between the human and the natural worlds (Palsson et al., 2013; Brondizio et al., 2016; Scheffer, 2020). Once the privileged domain of ecological and Earth sciences, the observation of how human processes alter and shape the biophysical and human environment has started to become a key topic within social science circles (Haberl et al., 2016; Gayo et al., 2022). Similarly, social science scholars have brought novel concerns to the table about the conditions allowing society to undertake actions to respond to or prevent such human-induced changes in the environment (Olsson et al., 2014; Lenton and Latour, 2018; Urquiza et al., 2021). The very idea of “Nature,” and of a separation between Humanity and Nature, has become a key topic of debate within social science practice (Escobar, 2011; Kelsen, 2014; Kadykalo et al., 2019).

While this has fostered a creative expansion of the analytical toolboxes employed to tackle socio-environmental transformations with novel concepts and methods (Micklin, 2019), it has been accompanied by a fragmentation of the topic into an array of different (and sometimes conflicting) approaches, each imbued with distinct conceptions of change and more or less explicit value-commitments (Feola, 2015; Fazey et al., 2018; Billi et al., 2021). While some scholars have emphasized that overcoming the global crises we are faced with requires structural and radical changes in the forms of production and socio-political structures, others have focused on critically observing the processes and conditions driving change at a systemic level, and others still have favored a more hands-on approach, discussing and actively fostering cognitive, cultural or agential enablers of change (Scoones et al., 2018, 2020). In addition, a fourth and more semiotic perspective has examined socio-historical changes within conceptions and descriptions of change and transformations and how these concur to challenge or reproduce established forms of societal organization (Patterson et al., 2017; Bennett et al., 2019).

With few -albeit notable- exceptions, each of these approaches has been pursued differently and in relatively self-enclosed epistemic communities, often reflecting enduring disciplinary or theoretical divides, and preventing a reflexive debate on the interactions and possible synergies between each of these perspectives on socio-environmental transformations (Victor, 2015; Billi et al., 2019). Moreover, the structural differences between disciplines, research institutions, and between the Global North and the Global South, tend to grant privileged visibility to, certain already established, perspectives, visions and methods; while others remain relatively invisible, despite their potential to provide a fresh view on the interdependence and reciprocal changes induced by society on the environment and the other way around (Santos, 2016; Reiter, 2020; Sapiains et al., 2021).

In order to overcome these traditional divisions and foster a more plural, creative and reflexive debate, this Research Topic (dossier) invited scholars and research practitioners from different regions and epistemic communities to engage with the different approaches to and dimensions of socio-environmental transformations, as well as their possible interaction and integration. Thus, we present the results of novel theoretical and methodical reflections on different conceptions of and analytical approximations of socio-environmental transformations, considering particular experiences and case studies.

Out of the six papers included in this Research Topic, two focus (Mascareño; Hert and Manville) on the dialogue between social and ecological systems, and other two (Büscher; Juri et al.) on that of social and technological systems. The remaining two (Hurlbert; Huang and Harvey) deal with conceptual and methodological issues related to the understanding of Climate crises and learnings meant to tackle said crises.

Mascareño shows a novel theoretical framework how Niklas Luhmann's theory of social systems can contribute to Marten Scheffer's critical transitions theory with a robust, complexity-based approach to social communication that can offer complementary forms of addressing social-ecological transitions. After highlighting the joint foundations of both theoretical frameworks in second-order cybernetics, and explaining Luhmann's positioning of meaning-making communication as the basic operation constituting social systems, he then reflects on the possibility of observing communication dynamics to provide both early warning signals of environmental problems and depictions of possible futures, crucial elements for refining our comprehension of social-ecological transitions. The author argues that the social-ecological transitions are an unresolvable mix of materiality and meaning. To that extent, a theoretical dialogue between the science of ecosystems and the science of society contributes to overcoming the nature/society divide and invites to think whether the era of the biosphere can manage the effects of the so-called Anthropocene era.

Along a similar avenue, Hertz and Mancilla's paper provides a novel approach to understanding the complex dynamics of the social-ecological system. Based on Barad's approach, the authors re-examine the paradigmatic case of the Baltic cod collapse in the eighties and pay attention to the material-discursive apparatus that produces a particular “cut” that provides a fresh angle for understanding sustainability problems. This article pursues two objectives: firstly, based on the empirical case, it questions the thick boundaries between conditions and causal elements that explain the processes in which social-ecological systems evolve, and discusses the hegemonic dominance of the material-discursive apparatus of modernity (where nature is relegated to the realm of the object as intrinsically purpose–and valueless) and the need to develop a different material-discursive apparatus to transform the socio-ecological systems; secondly, it provides a “transformative cut,” a playful environment, based on local knowledge, participatory processes, or arts-based methods, to imagine alternative pathways and provide ground for inspiring change.

Büscher's paper advances a theoretical reflection on the concept of “socio-technical” used in transition research. This scholarship has made a great contribution in highlighting the need for more integrative approaches to study change and innovation, embracing “social” elements (e.g., values, preferences, decision-making, and social organization) alongside purely technological ones. However, the interrelation between those technical and social elements is not always explicitly discussed, rather tending to be taken for granted. Building on the theoretical premises of “operative constructivism,” the author introduces the distinction between tight and loose coupling to distinguish predominantly deterministic, or “tightly” coupled relations, from eminently selective, or “loosely” coupled ones. He then reflects how both kinds of relations can operate as much on “technical” operations (i.e., energy and matter interchanges) and “social” ones (i.e., communications), depending on whether the emphasis is on ensuring control (tightly coupled technical and individual/organizational/societal action) or on exploring possibilities for change. The author concludes that this lens enriches the concept and practice of socio-technical transitions opening up new avenues for analysis.

On a more applied note, Juri et al. attempts to outline a framework that adopts and adapts the Transition Design approach to educational institutions and platforms in the context of Latin America. Transition Design is an emerging approach seeking to facilitate societal transition processes by supporting, connecting, or developing interventions to intentionally change values, framing, technologies, social practices, and infrastructures while reshaping interactions between socio-technical and socio-ecological systems. Putting in dialogue approaches emerged in the Global North and the Global South, the paper strives to expand the original TD framework, e.g., explicitly considering the relevance of the restructuring of governance, power relations, and empowerment while incentivizing new political capacities for transformation; or fostering openness to a multiplicity of worldviews and forms of knowledge, to promote a mutual learning space for transformation and change. Illustrating its reflection through the analysis of three cases, the paper puts forth a theoretical topography of what Transition Design workshops, or alternative pedagogical spaces, would need to engage with, integrate or expand within the region: a type of systemic intervention that aims to ensure pluriversality, solidarity, and mutual learning.

In a complementary avenue, the paper from Huang and Harvey proposes an emerging method to assess social learning in the context of large transdisciplinary research programs in Climate Change and Resilience, through a constructivist perspective on knowledge creation, emphasizing the central role program stakeholders must play in constructing and interpreting findings to achieve a robust understanding of complex social realities. By making explicit the programs' theory of change, it allows to distinguish between isolated change “events” (meetings, training, policies being adopted, etc.) and evolutions in deeper patterns, system structures, and mental models that emerge from social learning processes. This help creating more clarity on the program's narrative to help program managers, researchers, and evaluators to distill evidence-informed lessons that are transferable for future program design, as well as to better detect the underlying factors, such as structural barriers and existing norms, that foster or inhibit the processes and outcomes of social learning. In addition, given the perceived link between learning and transformation, this method may also provide insights on how we can better embed the processes, structures, and ways of learning into programs in ways that allow us to move away from incremental forms of change.

Finally, Hurblert's paper argues that problem framing in relation to complex problems is important for addressing climate change and driving policy change. Nevertheless, as the impacts of climate change accelerate and the window of opportunity closes for making transformative changes to address climate change, there has been increasing use of the “threat” frames including “Anthropocene,” “climate crisis,” “climate emergency,” and “climate catastrophe.” This paper argues that crisis frames are not enough to engender the transformative change required to address climate change (The Anthropocene) and often do not engender a sense of empowerment. Moreover, there is much we don't know about time pressure messaging, cognition, motivation to act, personal levels of knowledge, and decision-making heuristics. However, we do know that people cognizant of greater climate change risks, who are better informed, are more likely to support climate change policies. Therefore, the framing of climate change as a crisis without providing accompanying information about the ability to make changing and hope will likely polarize people and their beliefs and actions.

While engaging with different frameworks and problems, all these papers share a common interest for uncovering the conceptual, epistemological, and methodological ambiguities underlying both the study and practice of socio-environmental transformations, and offer novel analytical perspectives to overcome them and advance toward a more effective and genuinely transformative approach to research and action.

From these very timely and interesting contributions, it can be gathered the need and opportunity to strengthen the dialogue between different social science traditions and, even more importantly, between the social and the natural sciences. While the call for inter and transdisciplinary approaches on sustainability has been long raised in scholarship (Kates et al., 2001; Cornell et al., 2013) too few are the approaches that do justice to these needs, with social sciences often limiting themselves to timidly stand on the boundaries of the mainstream debates -often clutching to traditional concepts and disciplines proper of these disciplines- and “natural” science disciplines expanding to “colonize” the domain of the social sciences with approaches that provide broadness of scope but often lack enough depth (Olsson et al., 2015; Chernilo, 2017). In the face of these trends, the contributions proposed in this Research Topic offer a light on how it is truly possible to build a robust and profound interpenetration between the social and natural sciences: for that, it is not enough to just translate concepts and methods, but it is necessary to bring forward a thorough and self-conscious reflection on the epistemological, ontological, and normative assumptions behind these concepts and methods, which can build the foundation for new approaches truly “transcending” disciplines in the quest for understanding and promoting transformations to sustainability.

In turn, this reflection may also contribute to reshaping the traditional form in which we understand the relationship between society and nature. Old European philosophy was founded both epistemically and normatively on a strict separation between human society and nature. However, today this is becoming increasingly inaceptable both in epistemic, ontological, and normative terms. The interconnected character of human-societal and natural transformation, growing evidence of the social organization of nature and the natural conditioning of society, growing questioning of anthropocentric ethics, and the increasing acknowledgment of non-Western and non-anthropocentric forms of knowledge, all point toward the need for theoretically informed sociological approaches which can overcome, question or transcend the taken-for-granted but bifurcation between “man” and “nature,” and advance toward a new paradigm of more holistic -and self-reflexive- sociological inquiry. Interestingly, this idea was already formulated in the very times of Old Europe by thinkers such as Mill Stuart (1969) but tended to be ignored by mainstream theory within the social and natural sciences alike. Today, finally, we are making significant steps to overcome this inertia and advance toward a more creative and reflexive paradigm for sociology, no longer separated but rather integrated with the natural sciences. While far from offering a comprehensive answer to the complex issues related to this challenge, this Research Topic hopes to have at least set the stage and motivated the quest, for such an inquiry.

## Author contributions

MB and CZ were the main writers of the Editorial, DM revised and edited the manuscript for content and form. All authors read and approved the final version of the manuscript.

## Funding

This work was supported by Center for Climate and Resilience Research (CR)2, FONDAP 1510009, National Agency for Research and Development (ANID), Chile, and by Fondecyt Postdoctorado 3220447, National Agency for Research and Development (ANID), Chile.

## Conflict of interest

The authors declare that the research was conducted in the absence of any commercial or financial relationships that could be construed as a potential conflict of interest.

## Publisher's note

All claims expressed in this article are solely those of the authors and do not necessarily represent those of their affiliated organizations, or those of the publisher, the editors and the reviewers. Any product that may be evaluated in this article, or claim that may be made by its manufacturer, is not guaranteed or endorsed by the publisher.
